# Calcium Determines *Lactiplantibacillus plantarum* Intraspecies Competitive Fitness

**DOI:** 10.1128/aem.00666-22

**Published:** 2022-07-19

**Authors:** Annabelle O. Yu, Lei Wei, Maria L. Marco

**Affiliations:** a Department of Food Science & Technology, University of California, Davis, California, USA; University of Naples Federico II

**Keywords:** *Lactiplantibacillus plantarum*, *Lactobacillus plantarum*, food fermentation, teff, probiotics, lactic acid bacterial calcium, divalent cation, intraspecific diversity, aggregation, ICP-MS

## Abstract

The importance of individual nutrients for microbial strain robustness and coexistence in habitats containing different members of the same species is not well understood. To address this for Lactiplantibacillus plantarum in food fermentations, we performed comparative genomics and examined the nutritive requirements and competitive fitness for *L. plantarum* strains B1.1 and B1.3 isolated from a single sample of teff injera fermentation batter. Compared to B1.1 and other *L. plantarum* strains, B1.3 has a smaller genome, limited biosynthetic capacities, and large mobilome. Despite these differences, B1.3 was equally competitive with B1.1 in a suspension of teff flour. In commercially sourced, nutrient-replete MRS (cMRS) medium, strain B1.3 reached 3-fold-higher numbers than B1.1 within 2 days of passage. Because B1.3 growth and competitive fitness were poor in mMRS medium (here called mMRS), a modified MRS medium lacking beef extract, we used mMRS to identify nutrients needed for robust B1.3 growth. No improvement was observed when mMRS was supplemented with nucleotides, amino acids, vitamins, or monovalent metals. Remarkably, the addition of divalent metal salts increased the growth rate and cell yields of B1.3 in mMRS. Metal requirements were confirmed by inductively coupled plasma mass spectrometry, showing that total B1.3 intracellular metal concentrations were significantly (up to 2.7-fold) reduced compared to B1.1. Supplemental CaCl_2_ conferred the greatest effect, resulting in equal growth between B1.1 and B1.3 over five successive passages in mMRS. Moreover, calcium supplementation reversed a B1.3 strain-specific, stationary-phase, flocculation phenotype. These findings show how *L. plantarum* calcium requirements affect competitive fitness at the strain level.

**IMPORTANCE** Ecological theory states that the struggle for existence is stronger between closely related species. Contrary to this assertion, fermented foods frequently sustain conspecific individuals, in spite of their high levels of phylogenetic relatedness. Therefore, we investigated two isolates of *Lactiplantibacillus plantarum*, B1.1 and B1.3, randomly selected from a single batch of teff injera batter. These strains spanned the known genomic and phenotypic range of the *L. plantarum* species, and in laboratory culture medium used for strain screening, B1.3 exhibited poor growth and was outcompeted by the more robust strain B1.1. Nonetheless, B1.1 and B1.3 were equally competitive in teff flour. This result shows how *L. plantarum* has adapted for coexistence in that environment. The capacity for the single macronutrient calcium to restore B1.3 competitive fitness in laboratory culture medium suggests that *L. plantarum* intraspecies diversity found in food systems is fine-tuned to nutrient requirements at the strain level.

## INTRODUCTION

Lactic acid bacteria (LAB) are essential for the production of thousands of different types of fermented dairy, meat, and plant-based foods and beverages (1), where they can account for over 95% of all microbes present (2, 3). These bacteria are also inhabitants of the human, animal, and insect microbiomes (4). A common feature among LAB-associated habitats is that they are nutrient rich, frequently containing high quantities of sugars as well as other macro- and micronutrients (e.g., vitamins, amino acids, trace metals, and nucleotides). Consistent with this assertion, numerous studies have shown that LAB strains have undergone genomic reduction as a result of adapting to the environments in which they are found (e.g., food fermentations and mucosal surfaces) (5–9). Gene gain and loss likely occur as a function of the selective pressures occurring in food habitats (e.g., yogurt [10]). However, the extent of intraspecies variation among LAB within food fermentations and the consequences of strain differences on ecological robustness remain to be determined.

Lactiplantibacillus plantarum (formerly known as Lactobacillus plantarum) is one of the most frequently isolated LAB species from fresh and fermented foods (vegetables, fruits, dairy, and meat) and intestinal sources (11–13). Because of its capacity to colonize different plant and animal habitats and its genome plasticity, *L. plantarum* is regarded as a “generalist” or “nomadic” LAB (14–16). Strains of *L. plantarum* tend to have larger genome sizes (3 to 3.6 Mbp) than other LAB (1.5 to 3.0 Mbp) and fewer nutritional requirements (14, 17). However, as shown for the model strain WCFS1, *L. plantarum* still requires vitamins (d-pantothenate, d-biotin, nicotinic acid, and riboflavin) and amino acids (arginine, glutamic acid, glycine, leucine, methionine, tryptophan, and valine) for robust growth (18).

Recent comparative genomic analysis and phenotypic screening approaches have revealed the tremendous intraspecific variation in the *L. plantarum* species (14, 19, 20). We recently reported that *L. plantarum* isolates from the same (fermented) plant food type tend to use the same carbohydrates for growth and exhibit similar stress tolerance levels, indicating the presence of strong selective pressures for strain enrichment and adaptation within those environments (20). However, despite certain similarities, strain variation is not limited to isolation source, and we and others showed that *L. plantarum* recovered from the same food, plant, or animal environment can have very different genotypic and phenotypic properties (19). Although it is feasible that this conspecific diversity is important for sustaining *L. plantarum* within individual habitats, the traits needed for strain coexistence should be identified.

*L. plantarum* B1.1 and B1.3 were isolated from a single sample of teff injera and previously compared to each other and to other plant-associated *L. plantarum* strains (20). The carbohydrate utilization and stress tolerance capacities of strain B1.1 were robust and similar to those of reference strain NCIMB8826R and other *L. plantarum* isolates. Conversely, growth of strain B1.3 was poor on glucose and other sugars and B1.3 was also unable to tolerate acidic pH and high-salt, high-ethanol, and high-temperature conditions (20). To determine how *L. plantarum* B1.3 could be competitive in teff injera fermentations where other *L. plantarum* strains such as B1.1 are present, we investigated B1.3 and B1.1 for their genome characteristics, competitive fitness, and specific nutritional requirements for robust growth in nutrient-replete MRS laboratory culture medium. These studies led us to identify how the requirement for divalent metal cations and calcium specifically differs between strains of *L. plantarum* and how those nutrients are important for sustaining B1.1 and B1.3 in coculture.

## RESULTS

### *L. plantarum* B1.3 contains a large mobilome and lacks numerous biosynthetic pathways present in other *L. plantarum* strains.

The genome of *L. plantarum* B1.3 (3.09 Mbp) is approximately 111 kbp smaller than the genome of strain B1.1 (3.17 Mbp) (20). Compared to 663 *L. plantarum* genome assemblies, the genome size of strain B1.3 ranks in the lowest 10%. Consistent with its smaller size, the genome of *L. plantarum* B1.3 contains deletions ranging in size from 2,200 to 25,000 bp (see Table S1 in the supplemental material). These deletions are at different locations in the chromosome relative to the reference strain *L. plantarum* WCFS1 and frequently occur in gene loci coding for amino acid and vitamin biosynthetic pathways and transporters and for the production of cell-surface-associated and carbohydrate utilization proteins (Table S1). For example, B1.3 lacks genes required for the synthesis of thiamine, riboflavin, cystine, aromatic amino acids, and branched-chain amino acids (Table S1 and Fig. S1). By comparison, the genome composition of strain B1.1 is similar to that of other plant-associated *L. plantarum* strains and the model *L. plantarum* strain WCFS1 (21, 22).

The number of predicted proteins in individual Clusters of Orthologous Groups (COG) categories also differed between *L. plantarum* B1.3 and B1.1 (Table 1). Strain B1.1 contains higher numbers of gene clusters than B1.3 in the transcription (K), energy production and conversion (C), nucleotide (F), coenzyme (H), lipid (I), and inorganic ion (P) transport and metabolism COGs (Table 1). Strain B1.3 has more gene clusters than B1.1 for cell wall/membrane/envelope biogenesis (M), posttranslation modification/protein turnover/chaperone (O), replication/recombination/repair (L), and amino acid transport and metabolism (E) COGs. The greatest difference between the two strains, however, is in the mobilome COG. Strain B1.3 is annotated to contain 346 gene clusters, whereas B1.1 contains only 118, constituting nearly a 3-fold difference (Table 1). Over 90% of B1.3 genes in the mobilome COG are annotated as transposases, and the remainder are predicted to encode prophages or have a role in plasmid replication.

**TABLE 1:** Distribution of COG categories in *L. plantarum* B1.1 and B1.3

COG category^*a*^	Genome characteristic	No. of COGs in strain:	
		B1.1	B1.3
Cellular process and signaling	D: cell cycle control, cell division, chromosome partitioning	41	35
	M: cell wall/membrane/envelope biogenesis	134	153
	N: cell motility	15	16
	O: posttranslation modification, protein turnover, and chaperones	75	85
	T: signal transduction mechanism	125	117
	U: intracellular trafficking, secretions, and vesicular transport	17	14
	V: defense mechanism	76	84
	W: extracellular structures	3	3

Information storage and processing	J: translation, ribosomal structure, and biogenesis	208	201
	K: transcription	226	191
	L: replication, recombination, and repair	114	124

Metabolism	C: energy production and conversion	118	109
	E: amino acid transport and metabolism	177	201
	F: nucleotide transport and metabolism	92	85
	G: carbohydrate transport and metabolism	211	216
	H: coenzyme transport and metabolism	124	103
	I: lipid transport and metabolism	101	81
	P: inorganic ion transport and metabolism	104	97
	Q: secondary metabolites biosynthesis, transport, and catabolism	20	20

Other	R: general function prediction only	205	160
	S: function unknown	187	171
	X: mobile: prophages, transposons^*b*^	118	346
	None	581	509

a COG categories were assigned using Anvi’o (v6.1) using the program ‘anvi-run-ncbi-cogs’.

b The “X: mobile: prophages, transposons” COG is highlighted in gray to emphasize the 2.9-fold-higher number of COGs in this category in the B1.3 genome.

### *L. plantarum* B1.3 growth is restored in mMRS containing beef extract.

**FIG 1:**
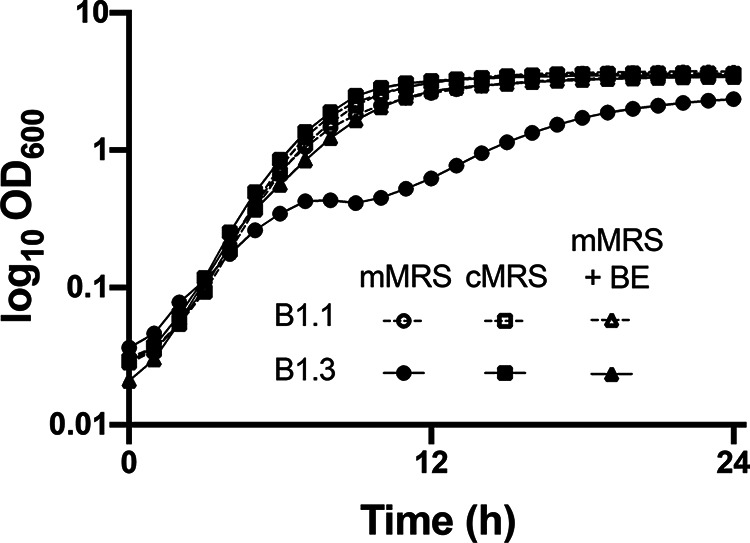
Growth of *L. plantarum* B1.3 is improved in mMRS supplemented with beef extract. *L. plantarum* B1.1 and B1.3 were inoculated into mMRS, cMRS, or mMRS supplemented with beef extract (BE; 8 g/L) and incubated at 30°C for 24 h. Results shown are representative average OD_600_ ± standard deviation from duplicate experiments with three independent cultures.

**FIG 2:**
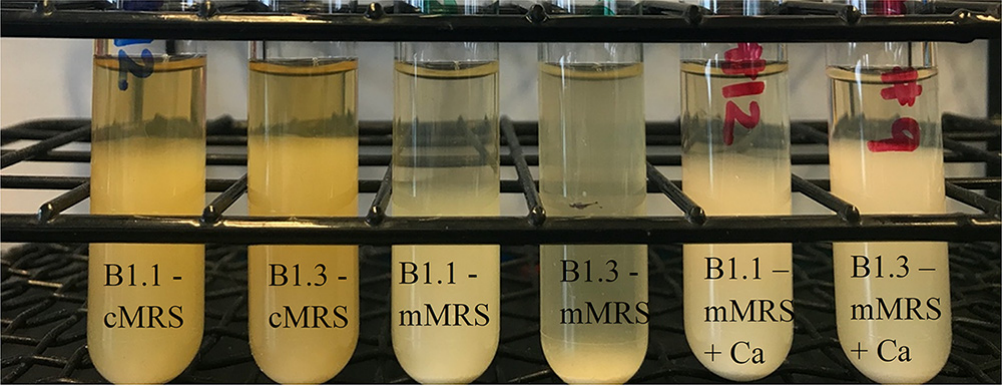
Autoaggregation of *L. plantarum* B1.1 and B1.3 in cMRS, mMRS, and mMRS supplemented with 5 mM CaCl_2_. *L. plantarum* B1.3 and B1.1 grown in triplicate were imaged after incubation at 30°C for 72 h. Representative cultures are shown.

Consistent with the reduced biosynthetic capacity of B1.3, that strain grew poorly in mMRS, a modified version of MRS medium lacking beef extract (Fig. 1) (see also reference 20). B1.3 exhibited biphasic growth in mMRS with lower growth rates (0.38 ± 0.01 h^−1^ and then 0.07 ± 0.01 h^−1^) than strain B1.1 (0.61 ± 0.01 h^−1^) (Fig. 1). Additionally, it was observed that within the first 24 h after stationary phase was reached, B1.3 but not B1.1 flocculated, forming aggregates (biofilm) which were deposited on the bottom of the test tube (Fig. 2).

Notably, these differences between B1.1 and B1.3 were not found when the strains were incubated in a commercially sourced MRS medium (cMRS) containing beef extract (Fig. 1 and 2). The growth rate of B1.3 in cMRS (0.61 ± 0.01 h^−1^) was not significantly different from that of B1.1 (0.61 ± 0.01 h^−1^) in the cMRS laboratory culture medium, nor was there evidence of flocculation (Fig. 2). The importance of beef extract (BE) for B1.3 growth in cMRS was confirmed by adding BE as a supplement into mMRS (mMRS-BE) (Fig. 1). In mMRS-BE, the growth rate of B1.3 (0.62 ± 0.01 h^−1^) was equal to that of B1.1 (0.63 ± 0.01 h^−1^) and was comparable to growth in cMRS. Furthermore, with the addition of BE, aggregation of B1.3 was no longer observed and instead the strain matched the profile seen when B1.3 was grown in cMRS (Fig. S2). These findings show that the robustness of B1.3 growth is dependent on a factor provided by the BE.

### Strain B1.3 outcompetes B1.1 in cMRS and teff flour.

**FIG 3:**
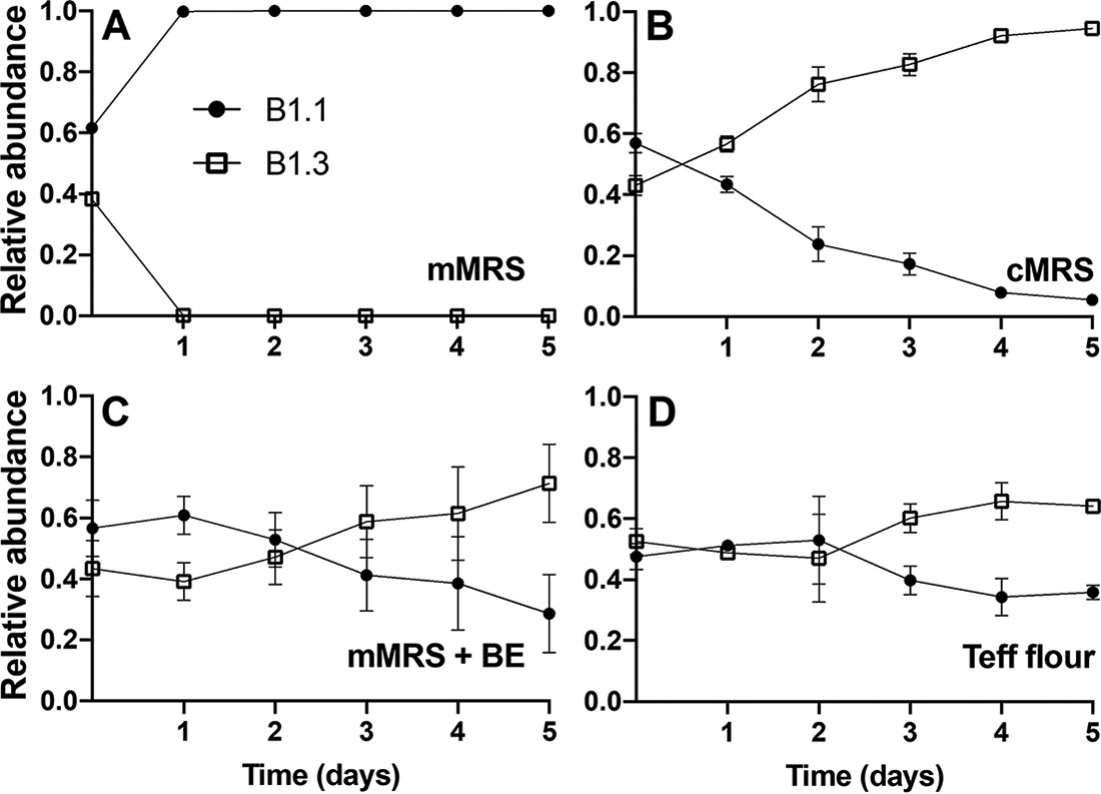
*L. plantarum* B1.3 exhibits a high level of competitive fitness compared to B1.1 in mMRS with beef extract (BE) and in teff flour. Equal numbers of *L. plantarum* B1.1 and B1.3 (10^5^ CFU/mL) were coinoculated in (A) mMRS, (B) cMRS, (C) mMRS supplemented with beef extract (BE; 8 g/L), or (D) teff flour mixed with PBS and incubated at 30°C for 24 h. A total of 50 μL was transferred into fresh medium (constituting 1% of the final volume) on each of the subsequent 5 days. Results shown are representative average OD_600_ ± standard deviation for duplicate experiments with three independent cultures.

We next measured the competitive fitness of B.1.3 and B1.1 in coinoculation assays in which the strains were inoculated in equal proportions and passaged daily in fresh culture medium for 5 consecutive days. As expected, based on growth rates, strain B1.3 was rapidly outcompeted by B1.1 in mMRS (Fig. 3A). B1.3 was no longer detectable (detection limit of 10^7^ CFU/mL) within 24 h of incubation in mMRS. Cell numbers of strain B1.1 increased from 1 × 10^5^ CFU/mL to 2 × 10^9^ CFU/mL during that time, reaching at least a 100-fold increase over B1.3 (Fig. S3). Conversely, B1.3 outcompeted B1.1 in cMRS (Fig. 3B) and in mMRS-BE (Fig. 3C). Within 2 days of sequential passage in cMRS, strain B1.3 outgrew B1.1 in a ratio of approximately three to one (Fig. 3B). Strain B1.3 reached higher numbers than B1.1 in mMRS-BE as well, although the increases were not significant (*P* < 0.05, Student’s *t* test) (Fig. 3B and Fig. S3).

To assess the capacity of B1.1 and B1.3 to compete under nutritive conditions resembling their isolation source, the two strains were incubated in an aqueous suspension of teff flour, the primary ingredient in teff flour injera batter. Strains B1.1 and B1.3 grew in the teff flour suspension and reached similar numbers after 24 h of incubation (starting at 10^5^ CFU/mL and reaching 4.6 × 10^8^ CFU/mL after 24 h at 30°C, approximately 4 generations) (data not shown). When coinoculated into the teff flour suspension, B1.1 and B1.3 were present in equal proportions after 24 h of incubation (Fig. 3D). However, by the 3rd day of passage, B1.3 reached higher numbers than B1.1, and the differences were sustained for subsequent days of passage (Fig. 3D and Fig. S3). These findings show that despite the smaller genome size and limited biosynthetic capacity of *L. plantarum* B1.3, that strain evolved for growth in nutrient-rich environments and B1.1 and B1.3 are adapted for coexistence in teff flour.

### *L. plantarum* B1.3 growth in mMRS is not improved with the addition of nucleotides, amino acids, or vitamins.

**FIG 4:**
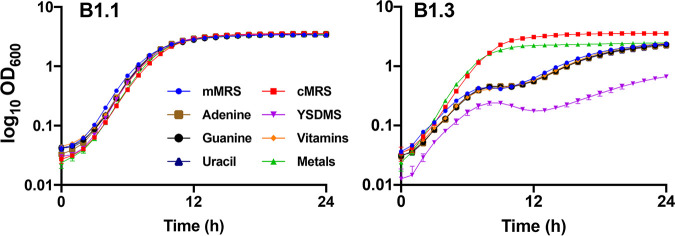
Growth of *L. plantarum* B1.1 and B1.3 in mMRS amended with different nutritional supplements. *L. plantarum* B1.1 and B1.3 were incubated at 30°C for 24 h in cMRS or mMRS containing additional adenine, guanine, or uracil; yeast synthetic dropout medium supplement; a mixture of vitamins; or a mixture of trace metals. Results shown are representative average OD_600_ ± standard deviation for duplicate experiments with three independent cultures.

Because the B1.3 genome lacks many biosynthetic pathways and nutrient transporters present in other *L. plantarum* strains and because beef extract and teff flour are replete with different amino acids, carbohydrates, nucleosides, vitamins, and trace metals, it was not possible to predict which nutrient(s) is most important for robust B1.3 growth. Therefore, we performed nutrient addition experiments with mMRS with the goal of identifying compounds able to improve growth of B1.3 in cMRS so that it is comparable to B1.1. These experiments showed that supplementation of exogenous nucleotides (adenine, guanine, and uracil) was not sufficient to increase the growth rate and cell yields of B1.3 in mMRS (Fig. 4 and Table 2). Growth was also not improved when amino acids were added to mMRS, either as single amino acid additions (Fig. S4) or when provided in a mixture with adenine, inositol, and *p*-aminobenzoic acid in yeast synthetic dropout medium supplement (YSDMS) (Fig. 4 and Table 2). Moreover, the growth rate of strain B1.3 was not altered in mMRS supplemented with a vitamin mixture containing biotin, folic acid, pyridoxine hydrochloride, thiamine HCl, riboflavin, nicotinic acid, d-pantothenate, vitamin B_12_, *p*-aminobenzoic acid, and thioctic acid (Fig. 4 and Table 2). For *L. plantarum* B1.1, growth of that strain was not affected in mMRS supplemented with nucleotides or amino acids (Fig. 4, Table 2, and Fig. S4). The addition of the vitamin mixture had a negative effect on B1.1, resulting in a reduced growth rate compared to that when the strain was incubated in mMRS (0.53 ± 0.01 h^−1^) (*P* < 0.05, Student’s *t* test) (Table 2).

**TABLE 2:** Growth rates of *L. plantarum* B1.1 and B1.3 in MRS supplemented with different nutrients^*a*^

Supplement	Avg growth rate (h^−1^) ± SD of strain:	
	B1.1	B1.3
mMRS	0.59 ± 0.01	0.40 ± 0.01
cMRS	0.59 ± 0.01	**0.59 ± 0.02**
Adenine	0.60 ± 0.01	0.41 ± 0.01
Guanine	0.57 ± 0.02	0.41 ± 0.01
Uracil	0.59 ± 0.01	0.42 ± 0.01
YSDMS^*b*^	0.61 ± 0.01	0.40 ± 0.04
Vitamins^*c*^	**0.53 ± 0.01**	0.39 ± 0.01
Metals^*d*^	0.61 ± 0.02	**0.59 ± 0.01**
NaCl	0.57 ± 0.01	0.37 ± 0.06
MgSO_4_	0.54 ± 0.01	**0.42 ± 0.03**
AlK_2_O_4_	0.60 ± 0.02	0.36 ± 0.01
KCl	0.57 ± 0.01	0.37 ± 0.02
CaCl_2_	0.62 ± 0.06	**0.60 ± 0.01**
MnSO_4_	0.64 ± 0.04	**0.47 ± 0.01**
FeSO_4_	0.59 ± 0.02	**0.47 ± 0.02**
CoSO_4_	0.59 ± 0.02	**0.38 ± 0.00**
NiSO_4_	0.60 ± 0.01	**0.47 ± 0.00**
CuSO_4_	0.57 ± 0.02	**0.46 ± 0.00**
ZnSO_4_	**0.39 ± 0.00**	0.38 ± 0.02
Na_2_MoO_6_	0.56 ± 0.01	**0.39 ± 0.01**

a The average growth rate ± standard deviation for three individual replicates is shown. Bold type indicates significantly different (*P* < 0.05) growth rates compared to incubation in mMRS (shaded in gray) according to an unpaired, two-tailed Student *t* test.

b mMRS was supplemented with 0.96 g/L of yeast synthetic dropout medium supplement (YSDMS) containing all 20 standard amino acids, adenine, inositol, and *p*-aminobenzoic acid.

c The vitamin supplement contained 16.3 μM biotin, 9.0 μM folic acid, 97.3 μM pyridoxine hydrochloride, 29.6 μM thiamine HCl, 26.8 μM riboflavin, 81.2 μM nicotinic acid, 21.0 μM calcium d-pantothenate, 147 nM vitamin B_12_, 72.9 μM *p*-aminobenzoic acid, and 48.5 μM thioctic acid.

d mMRS was supplemented with a mixture of the following trace metals (each at a concentration of 0.5 mM): NaCl, KCl, MgSO_4_, CaCl_2_·2H_2_O, MnSO_4_·H_2_O, FeSO_4_·7H_2_O, CoSO_4_·7H_2_O, NiSO_4_·6H_2_O, CuSO_4_·5H_2_O, and ZnSO_4_·7H_2_O.

### Metal salts improve *L. plantarum* B1.3 growth in mMRS.

The growth rate of *L. plantarum* B1.3 increased significantly in mMRS containing a mixture of metal salts (0.5 mM concentrations each of the following: NaCl, MgSO_4_, KCl, CaCl_2_, MnSO_4_, FeSO_4_, CoSO_4_, NiSO_4_, CuSO_4_, and ZnSO_4_) (*P* < 0.05, Student’s *t* test) and was similar to that of B1.3 in cMRS (Table 2 and Fig. 4). This effect was specific for B1.3 because the growth rate and cell numbers of B1.1 were not altered by adding metal salts to the laboratory culture medium (Table 2 and Fig. 4).

**FIG 5:**
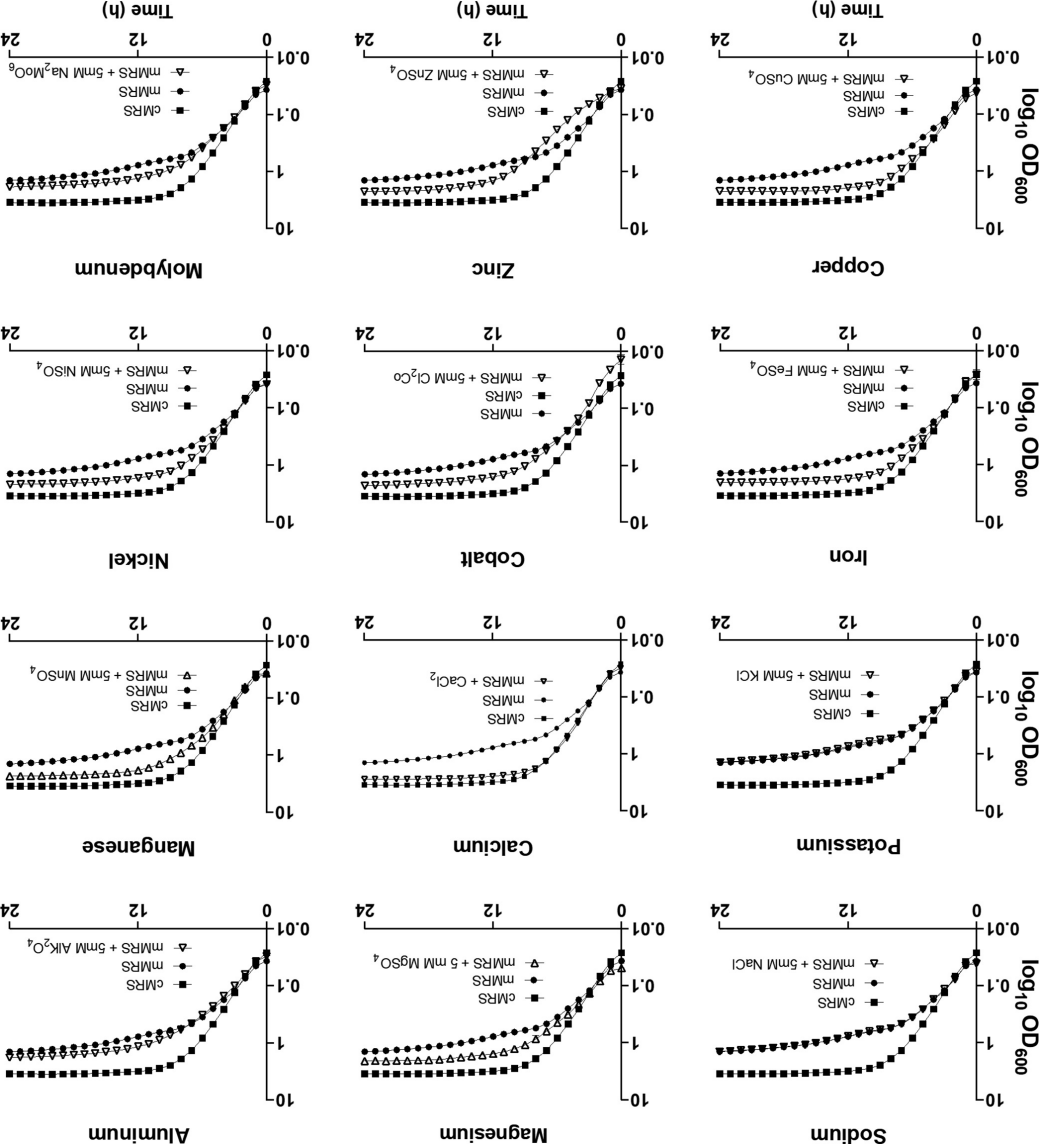
Growth of *L. plantarum* B1.3 in mMRS supplemented with trace metals. *L. plantarum* B1.3 was inoculated into mMRS containing 5 mM individual trace metals and incubated at 30°C for 24 h. Results shown are representative average OD_600_ ± standard deviation for duplicate experiments with three independent cultures.

To assess the contributions of individual metals to *L. plantarum* growth, single-metal addition growth experiments were carried out in mMRS. Neither NaCl nor KCl was sufficient to improve the growth characteristics of B1.3 at either 5 mM (Table 2 and Fig. 5) or higher concentrations (50 mM or 100 mM) (data not shown). Although supplementing 5 mM AlKS_2_O_4_ resulted in significantly higher B1.3 cell numbers (indicated by the optical density at 600 nm [OD_600_] after 24 h of incubation; *P* < 0.05, Student’s *t* test) (Fig. 5), the growth rate of that strain in mMRS with the aluminum salt was not changed from that in mMRS (Table 2). Instead, the growth rates and final OD_600_ values of B1.3 were significantly higher during incubation in mMRS containing 5 mM concentrations of the divalent metal salts MgSO_4_, CaCl_2_, MnSO_4_, FeSO_4_, CoSO_4_, NiSO_4_, CuSO_4_, ZnSO_4_, or Na_2_MoO_6_ (*P* < 0.05, Student’s *t* test) (Table 2 and Fig. 5). Inclusion of those compounds also eliminated the diauxic growth of B1.3 in mMRS (Fig. 5).

Among the metal salt amendments, 5 mM CaCl_2_ yielded the greatest improvement in B1.3 growth in mMRS (Table 2 and Fig. 5). Surprisingly, this single modification improved the growth rate of B1.3 in mMRS (0.60 ± 0.01 h^−1^) to the extent that it was equal to the growth rate of this strain in cMRS (0.62 ± 0.01 h^−1^). This change occurred without increasing final cell numbers (final OD_600_ in mMRS-Ca, 3.06 ± 0.03, compared to that in cMRS, 3.60 ± 0.03, or mMRS-BE, 3.36 ± 0.05). Improved fermentation metabolism of B1.3 was also evident by the lower pH reached in mMRS with CaCl_2_ (pH 3.56) than in mMRS (pH 3.64). This effect was specific for B1.3 because the final pH was not affected for B1.1 when CaCl_2_ was added (pH 3.58 in mMRS with 5 mM CaCl_2_; pH 3.60 in mMRS). Notably, the increased growth rate of B1.3 in mMRS containing CaCl_2_ was not due to increased concentrations of the chloride or sulfate anions, as shown by their lack of effect for Mg^2+^ and Mn^2+^ salts and when either NaCl or KCl was added to the medium (Fig. S6). For *L. plantarum* B1.1, the addition of the individual metal salts in mMRS either did not change or reduced (5 mM ZnSO_4_) the growth rate of that strain (Table 2 and Fig. S5 and S7).

### Intracellular metal concentrations are reduced in *L. plantarum* B1.3.

**FIG 6:**
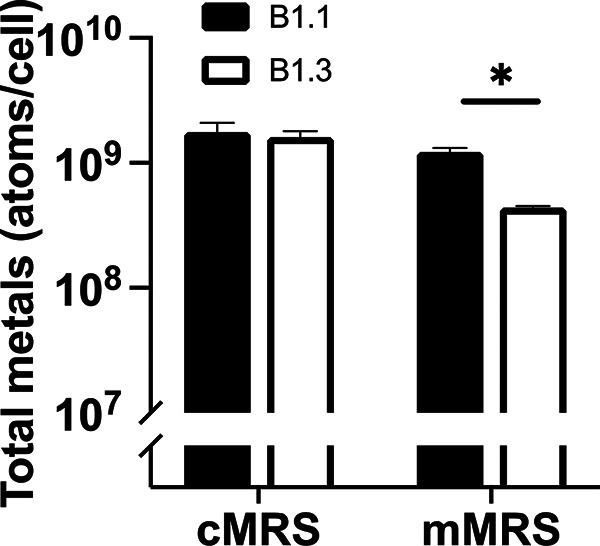
Sum of intracellular metal concentrations in *L. plantarum* B1.1 and B1.3 in cMRS or mMRS as determined by ICP-MS. Intracellular metal concentrations after growth in cMRS and mMRS are shown. Total metal concentrations were calculated by adding the concentrations of sodium, magnesium, aluminum, potassium, calcium, manganese, iron, copper, and zinc atoms. Levels of cobalt, nickel, and molybdenum were below the detection limit for ICP-MS. The average ± standard deviation for three replicate cultures is shown. Significant differences were calculated using a Student *t* test (*P* < 0.05).

To better understand why growth of strain B1.3 was improved in mMRS following the addition of divalent metal cations, intracellular metal concentrations for early-stationary-phase B1.1 and B1.3 cells grown in cMRS and mMRS were quantified by inductively coupled plasma mass spectrometry (ICP-MS). Intracellular sodium, magnesium, aluminum, potassium, calcium, manganese, iron, copper, and zinc metal concentrations were detectable above background levels by ICP-MS. Cobalt, molybdenum, and nickel were below the detection limit (data not shown). ICP-MS showed that in cMRS the sums of those metal concentrations were equivalent between B1.1 and B1.3 (Fig. 6). In mMRS, B1.1 contained 2.7-fold-higher levels than B1.3 (Fig. 6).

**FIG 7:**
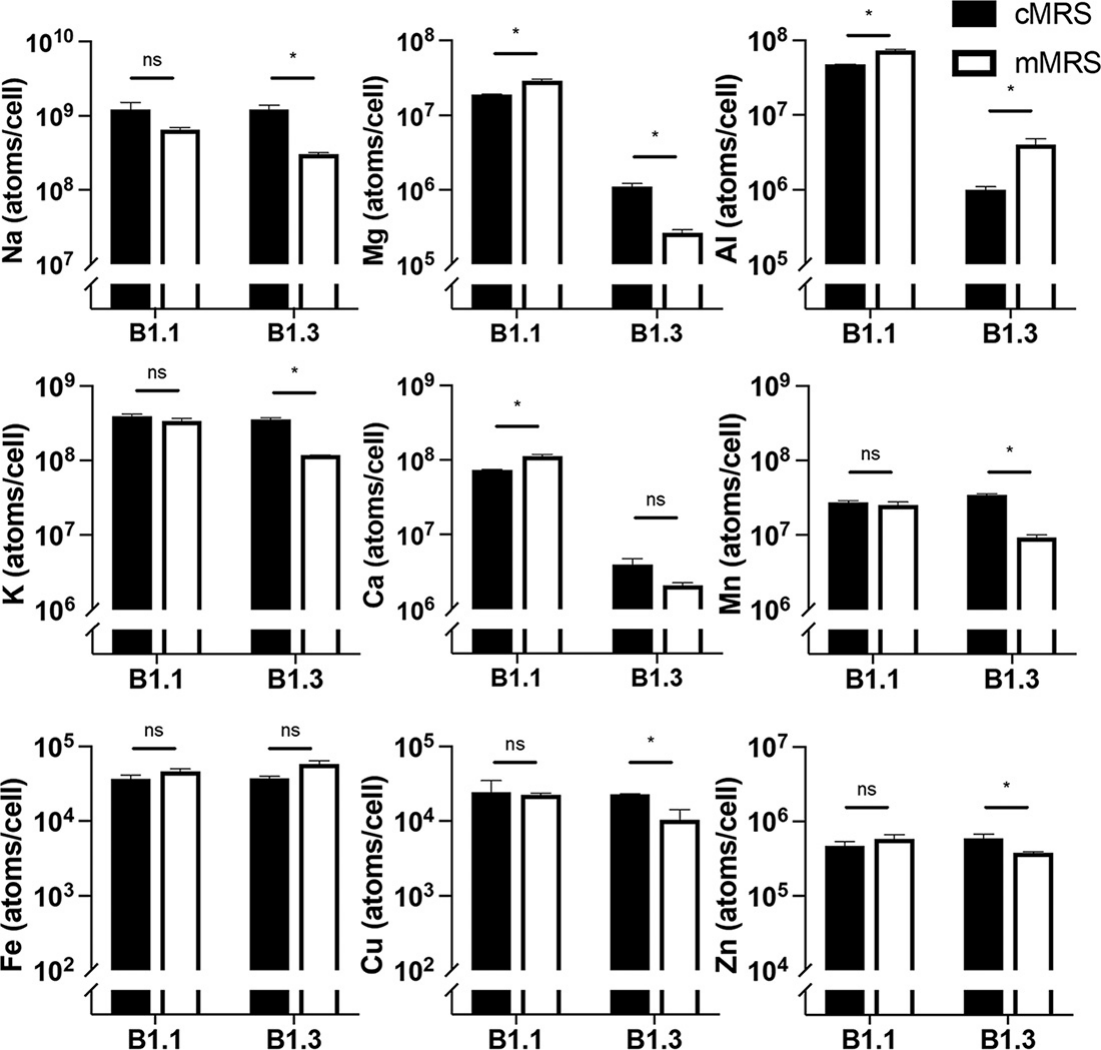
Intracellular metal concentrations in *L. plantarum* B1.1 and B1.3 in cMRS or mMRS as determined by ICP-MS. Intracellular metal concentrations of B1.1 and B1.3 after 24 h of growth in cMRS and mMRS are shown as determined by ICP-MS. The average ± standard deviation for three replicate cultures is shown. Significant differences were calculated using a Student *t* test (*P* < 0.05). ns, not significant.

Sodium and potassium were the most abundant metals in *L. plantarum* B1.1 and B1.3 in both mMRS and cMRS (between 5 × 10^8^ and 1 × 10^9^ atoms/cell) (Fig. 7). Within the range of detection, copper and iron were the least abundant (between 1 × 10^4^ and 7 × 10^4^ atoms/cell) (Fig. 7). MRS growth medium type did not affect the intracellular metal concentrations of strain B.1.1 (Fig. 7). As expected, based on total intracellular metal concentrations (Fig. 6), B1.3 contained significantly lower quantities of sodium, magnesium, potassium, calcium, manganese, copper, and zinc when grown in mMRS than when grown in cMRS (Fig. 7) (*P* < 0.05, Student’s *t* test). However, aluminum was increased (Fig. 7).

Comparisons of intracellular metal concentrations between the *L. plantarum* B1.1 and B1.3 strains showed that in cMRS *L. plantarum* B1.3 contained less aluminum (48-fold), calcium (18-fold), and magnesium (17-fold) and larger amounts of manganese (1.5-fold) than strain B1.1 (Fig. 7). In mMRS, eight out of the nine individual metals were present in significantly lower quantities in B1.3 than in B1.1, with magnesium (110-fold), calcium (55-fold), and aluminum (18-fold) constituting the greatest difference from B1.1 (Fig. 7). These results show that *L. plantarum* B1.3 and B1.1 have different capacities to transport and retain metals in the cell and that intracellular metal concentrations are culture medium dependent.

### *L. plantarum* B1.3 exhibits robust growth and competitive fitness in mMRS amended with supplemental calcium.

**FIG 8:**
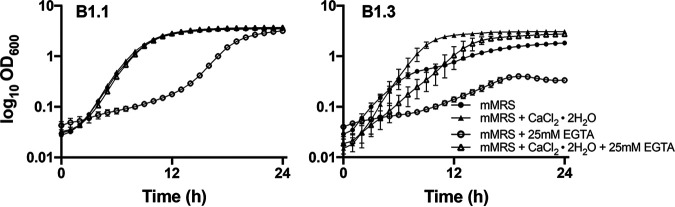
Growth of *L. plantarum* B1.3 is significantly impacted by the addition of EGTA, a metal chelator, but addition of calcium chloride ameliorates that effect. *L. plantarum* B1.1 and B1.3 were inoculated into mMRS or mMRS supplemented with 5 mM CaCl_2_·2H_2_O with or without 25 mM EGTA and were incubated at 30°C for 24 h. Results shown are representative average OD_600_ ± standard deviation for duplicate experiments with three independent cultures.

The robust growth of *L. plantarum* B1.3 in mMRS containing supplemental 5 mM CaCl_2_ and the low quantities of calcium detected in that strain in cMRS and mMRS led us to investigate the specificity for this calcium requirement. First, we found that B1.3 growth was not further improved when YSDMS was added to mMRS containing 5 mM CaCl_2_ (Fig. 4). Second, growth rates of B1.3 and B1.1 in mMRS were impaired when a metal chelator with a high affinity for Ca^2+^ (EGTA) was included. These effects occurred in an EGTA concentration-dependent manner (5 mM, 10 mM, and 25 mM EGTA) (Fig. S8) and were prevented (B1.1) or minimized (B1.3) when 5 mM CaCl_2_ was also included in the mMRS (Fig. 8). Third, stationary-phase B1.3 cells did not flocculate and instead remained suspended in the CaCl_2_-amended mMRS (Fig. S9). This reduction in flocculation exhibited by B1.3 was also observed under strict anaerobic conditions (data not shown) and occurred in the presence of either 2.5 mM or 5 mM CaCl_2_ (Fig. S9). Intermediate levels of flocculation occurred in mMRS with 1 mM CaCl_2_, whereas no change was observed in mMRS with 0.5 mM CaCl_2_ (Fig. S9). This effect was specific for calcium, because B1.3 cell aggregates formed when that strain was grown in mMRS containing MgSO_4_, AlK_2_O_4_, or NaCl (Fig. S10). By comparison, *L. plantarum* B1.1 remained suspended under all mMRS conditions (Fig. S11).

**FIG 9:**
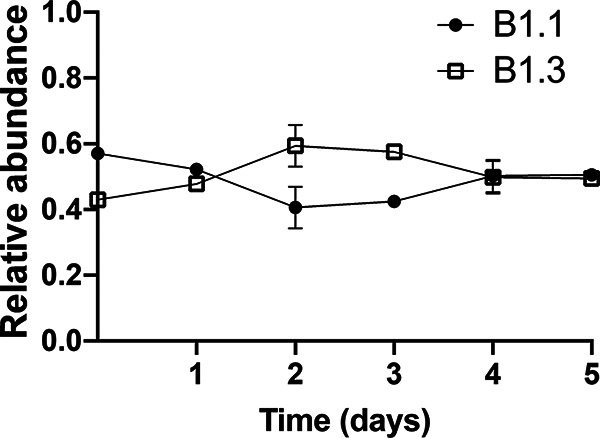
Competitive growth of *L. plantarum* B1.1 and B1.3 in mMRS amended with 5 mM CaCl_2_·2H_2_O. Equal numbers of *L. plantarum* B1.1 and B1.3 (10^5^ CFU/mL) were coinoculated in mMRS supplemented with 5 mM CaCl_2_·2H_2_O and incubated at 30°C for 24 h. A total of 50 μL was transferred into fresh medium (constituting 1% of the final volume) on each of the subsequent 5 days. Results shown are representative average OD_600_ ± standard deviation for duplicate experiments with three independent cultures.

Lastly, we examined the effect of calcium on the competitive fitness of B1.1 and B1.3 in coculture in mMRS supplemented with 5 mM CaCl_2_. Consistent with the improved growth of B1.3 in that laboratory culture medium, cell numbers of B1.3 and B1.1 were sustained in equal ratios (Fig. 9). The numbers of B1.3 were also significantly higher than those of B1.1 after 3 days of successive passage (*P* < 0.05, Student’s *t* test). In mMRS containing either 5 mM NaCl, 5 mM MgSO_4_, or 5 mM AlK_2_O_4_, B1.3 was outcompeted by B1.1 (Fig. S12). Our findings therefore confirm that sustaining both B1.3 and B1.1 in coculture is dependent on calcium availability.

## DISCUSSION

Food fermentations are complex microbial ecosystems which frequently contain multiple, highly related species and strains under constant selective pressure due to the changing physicochemical conditions in the food matrix. The cooccurrence of highly related phylogenetic clusters is not well understood but presumably is affected by both biotic and abiotic niche partitioning as well as mutualistic and antagonistic interactions (23, 24). These relationships are dependent on the prevailing nutrients in the food environment. In this study, we characterized *L. plantarum* strains B1.1 and B1.3 isolated from teff flour injera batter. These strains are genetically distinct, and due to its smaller size and large mobilome, strain B1.3 appears to have undergone genome reduction, potentially a consequence of adaptation to teff plants, flour, or injera fermentation batter. However, despite the growth impairments in mMRS, B1.3 was sustained in higher numbers than the more robust strain B1.1 in teff flour and outcompeted B1.1 by a ratio of three to one in cMRS. By examining the growth requirements of those strains, we identified the importance of calcium for competitive fitness of *L. plantarum* B1.3.

Because *L. plantarum* B1.1 and B1.3 were randomly selected colony isolates from a single sample of teff injera batter, we expected that they would exhibit similar genotypes and growth characteristics. Instead, these strains spanned the range of known features of the *L. plantarum* species. Whereas the genome content of B1.1 is similar to that of the model strain *L. plantarum* WCFS1 and other plant-associated LAB (20), B1.3 contains a highly reduced genome lacking genes coding for the synthesis of amino acids and vitamins and metabolism of a variety of carbohydrates. As for other LAB, genome rearrangements in B1.3 appear to have been the result of the acquisition of transposases and other mobile element-mediated gene disruptions (5). The limited biosynthetic capacity of B1.3 is consistent with the evolution of the *Lactobacillales* toward gene loss and metabolic simplification (5, 8, 13, 15–17). The selective pressures in teff flour may be particularly strong for genome reductions, as teff flour has higher concentrations of amino acids and vitamins than other cereal grains (barley, wheat, rye, maize, brown rice, sorghum, and pearl millet) (25).

*L. plantarum* B1.3 also grew poorly in mMRS, a modified version of MRS lacking beef extract. Because beef extract contains undefined concentrations of mono- and disaccharides, we previously measured carbohydrate preferences and stress tolerance characteristics of B1.3, B1.1, and other plant-associated *L. plantarum* strains, using mMRS as our reference medium (20). Despite the limitations of using mMRS for assessments of B1.3, differences between B1.3 and B1.1 in mMRS and cMRS provided an opportunity to identify the nutrients upon which B1.3 is particularly reliant. Remarkably, the provision of additional amino acids, nucleotides, and vitamins did not improve the growth of B1.3 in mMRS. The lack of effect in that medium may be due to the fact that mMRS is otherwise nutrient replete, containing undefined concentrations of those compounds in yeast extract and enzymatic digests of animal proteins (26).

Only with the addition of divalent metal salts to mMRS, either singly or combined, was there an improvement in *L. plantarum* B1.3 growth. The growth rates of B1.3 in mMRS with these elevated metal concentrations increased to levels similar to those found in cMRS and cMRS-BE and for B1.1. The differences in cation metal requirements between B1.3 and B1.1 were similarly confirmed by ICP-MS, showing that B1.3 cells contain significantly lower metal concentrations in mMRS. The altered abundance of intracellular metals between the two strains was not limited to the mMRS culture medium. Despite having similar total quantities of metals in cMRS, B1.3 still contained smaller amounts of magnesium, aluminum, and calcium than did strain B1.1. Presently, aside from a few reports suggesting different metal requirements for lactobacilli (27–31), the importance of metals for LAB growth and ecological interactions is not well understood. *L. plantarum* manganese requirements have been specifically investigated (30, 32, 33), likely because *L. plantarum* contains elevated levels of Mn^2+^ compared to other bacteria and because this element is known to confer resistance against oxidative (34, 35) and heat (36) stress. However, the potentially overlapping effects of metal cations (37) on *L. plantarum* and other lactobacilli remain to be determined. Although *L. plantarum* genomes contain a variety of metal transporters as well as enzymes with requirements for specific metals (21, 22), these pathways are not sufficiently defined to identify the specific genes required for metal homeostasis in the *L. plantarum* strains examined here.

Surprisingly, supplementing mMRS with CaCl_2_ resulted in the highest growth rate of B1.3 and enabled that strain to outcompete strain B1.1. Calcium is essential for intracellular signaling and regulation of multiple cellular processes including cell division and development, motility, stress response, and host-pathogen interactions (38, 39). For example, calcium ions were found to be stabilizing factors needed for the proper folding of manganese catalase in *L. plantarum* (40). However, because the addition of calcium in mMRS prevented the flocculation of *L. plantarum* B1.3 during stationary phase, the requirement for calcium may have been due to calcium-dependent effects on cell surface composition. To this regard, calcium and magnesium are required for bacterial cell wall and teichoic acid stability (41). Calcium was also shown to be important for aggregation, biofilm formation, and adhesion in both Gram-negative (e.g., Xylella fastidiosa and *Vibrio*) (42–44) and Gram-positive (e.g., Bacillus subtilis and Mycobacterium smegmatis) (41, 43, 45, 46) bacteria. In yeast, calcium regulates a flocculation phenotype in a lectin-specific manner (47). Although the specific role of calcium for B1.3 and B1.1 in teff flour fermentations remains to be determined, teff grains also have approximately 3-fold-higher levels of calcium (165.2 mg per 100 g teff) than sorghum, the cereal grain with the next highest quantities of calcium (50 mg per 100 g sorghum) (25). This nutritional requirement of B1.3 for calcium therefore provides further evidence of the adaptation of this strain to teff-associated habitats. Moreover, because both strains were inhibited when EGTA, a chelator with a high affinity for Ca^2+^, was included in the medium, these findings also indicate the importance of calcium for *L. plantarum* more generally.

In conclusion, by examining the nutritional requirements of B1.3, we identified the importance of divalent metal ions and particularly Ca^2+^ in the ecological fitness of *L. plantarum*. Because metals cannot be synthesized or degraded and may damage the cell if present in high quantities (48, 49), tight control over metal homeostasis is likely required by this species as well as other LAB in nutrient-rich food fermentations. These findings improve our understanding of the coexistence of multiple strains of the same microbial species within a single ecosystem as well as emphasize the importance of environmental context when evaluating inter- and intraspecies diversity and functionality of LAB in food fermentations.

## MATERIALS AND METHODS

### Bacterial strains and growth conditions.

*L. plantarum* strains B1.1 and B1.3 were previously isolated from Ethiopian teff injera (20) and were maintained as frozen glycerol stocks at −80°C. *L. plantarum* was routinely grown at 30°C in a commercial preparation of MRS (cMRS) (BD, Franklin Lakes, NJ), a modified MRS (26) (mMRS) prepared as lacking beef extract, mMRS supplemented with beef extract (8 g/L) (mMRS-BE), or a teff flour medium. To prepare the teff flour medium, whole-grain teff flour (Bob’s Red Mill, Milwaukie, OR) was sterilized by autoclaving for 1 h and then drying for approximately 15 h at room temperature. Teff flour medium was prepared by mixing the teff flour with phosphate-buffered saline (PBS; 137 mM NaCl, 2.7 mM KCl, 4.3 mM Na_2_HPO_4_·7H_2_O, 1.4 mM KH_2_PO_4_) (pH 7.2) at a dough yield [100 × weight (flour + PBS)/weight (flour)] (50) of 300. pH values were measured after 72 h of incubation in mMRS or mMRS with 5 mM CaCl_2_ using a Mettler Toledo pH probe (Mettler Toledo, Greifensee, Switzerland).

### Genome comparisons of B1.3.

Assembled and annotated genome sequences of *L. plantarum* B1.1 (WWCZ00000000) and B1.3 (WWCY00000000) were retrieved from the National Center for Biotechnology Information (https://www.ncbi.nlm.nih.gov/). Comparative genomics analysis using PATRIC was utilized to identify differences in the presence and absence of protein families compared to seven strains of plant-associated *L. plantarum* (1B1, K4, 8.1, AJ11, BGM37, EL11, and WS1.1) (20) and model strain WCFS1 (21) (https://www.patricbrc.org). Functional genomics analysis was preformed using Anvi’o 6.1 to cluster genes with similar functions and categorize them into different functional categories (51). Genome size comparisons were made against the 663 genome assemblies available in the National Center for Biotechnology Information database in January 2022 (https://www.ncbi.nlm.nih.gov/).

### Competitive fitness between *L. plantarum* B1.1 and B1.3 in different laboratory media and teff flour.

*L. plantarum* B1.1 and B1.3 were first incubated in cMRS for 24 h at 30°C. The cells were then collected by centrifugation at 5,000 × *g* for 5 min, washed twice in PBS (pH 7.2), and then suspended in cMRS, mMRS, mMRS supplemented with 5 mM CaCl_2_·2H_2_O or teff flour medium at a starting density of 10^5^ CFU/mL and grown for 24 h at 30°C. Cultures were then propagated at 1% into fresh medium every 24 h to simulate back-slopping. Serial dilutions of cultures after 24 h were plated on mMRS containing 2% (wt/vol) (58 mM) sucrose (mMRS-sucrose) and incubated for 48 h at 30°C. B1.1 and B1.3 colony size and shape on mMRS-sucrose were used to identify the two strains as shown in Fig. S13 in the supplemental material.

### *L. plantarum* growth requirements in mMRS.

*L. plantarum* B1.1 and B1.3 were incubated in cMRS for 24 h at 30°C, collected by centrifugation at 5,000 × *g* for 5 min, washed twice in PBS (pH 7.2), and then suspended in mMRS. The cell suspensions were then distributed into 96-well microtiter plates (Thermo Fisher Scientific, Waltham, MA) at an optical density at 600 nm (OD_600_) of 0.02. Wells contained mMRS supplemented with exogenous nucleobases (adenine, guanine, or uracil), vitamins, amino acids, or metals. Controls included mMRS diluted with an equal volume of water (5% [vol/vol]) instead of nutritional supplements. To avoid edge-effects in the microtiter plate, an antifog solution (0.1% Triton X-100 in 70% ethanol) was applied to the lid and the plates were sealed with Parafilm (Bemis, Shirley, MA). Growth was measured using a Synergy 2 microplate reader (BioTek, Winooski, VT) at 30°C every hour for 48 h. These experiments were conducted at least twice for each condition. Growth rates were calculated using the formula μ = (ln[OD_6002_/OD_6001_])/(T_2_ − T_1_) wherein the T_1_ was selected 1 h after the start of exponential growth. For experiments with the nucleobases, mMRS was amended with purines (adenine [148 μM] and guanine [132 μM]) or the pyrimidine (uracil [178 μM]). For incubation in a supplemental vitamin mixture, mMRS was amended to a final concentration of 16.3 μM biotin, 9.0 μM folic acid, 97.3 μM pyridoxine hydrochloride, 29.6 μM thiamine HCl, 26.8 μM riboflavin, 81.2 μM nicotinic acid, 21.0 μM calcium d-pantothenate, 147 nM vitamin B_12_, 72.9 μM *p*-aminobenzoic acid, and 48.5 μM thioctic acid.

For assessments of growth with supplemental amino acids, mMRS was amended with yeast synthetic dropout medium supplement (YSDMS) (0.96 g/L) (Sigma-Aldrich, St. Louis, MO) or Casamino Acids (BD, Franklin Lakes, NJ) (15 mg/mL). YSDMS contains all 20 amino acids, adenine, inositol, and *p*-aminobenzoic acid. For the single-addition amino acids, the concentrations of amino acids provided were five times the concentration described previously (18), with the exception of tyrosine, which was supplemented at the previously described concentrations (Teusink et al. [18]) due to its limited solubility in the growth medium.

To assess the effects of added metal salts, the following metal salts were used: CaCl_2_·2H_2_O, K_2_SO_4_·MgSO_4_, MnSO_4_·H_2_O, NaCl, Na_2_MoO_6_, NiSO_4_·6H_2_O, and ZnSO_4_·7H_2_O (Fisher Scientific, Pittsburgh, PA); AlK_2_O_4_, Cl_2_Mg·6H_2_O, Cl_2_Mn·4H_2_O, KCl, and Na_2_SO_4_ (Sigma-Aldrich, St. Louis, MO); FeSO_4_·7H_2_O (Spectrum, New Brunswick, NJ); CoSO_4_·7H_2_O (Acros Organics, Geel, Belgium); and CuSO_4_·5H_2_O (VWR International, Radnor, PA).

### ICP-MS.

*L. plantarum* B1.1 and B1.3 were incubated in cMRS and mMRS for 24 h at 30°C. Approximately 10^9^ cells were collected by centrifugation at 5,000 × *g* for 5 min and washed twice in PBS. The resulting cell material was digested by incubation at 95°C for 45 min in a 60% concentrated trace-metal-grade HNO_3_. The digests were then diluted with water to a final concentration of 6% HNO_3_ and analyzed with an Agilent 7500Ce ICP-MS instrument (Agilent Technologies, Palo Alto, CA) for simultaneous determination of select metals (Na, Mg, Al, K, Ca, Mn, Fe, Co, Ni, Cu, and Zn) at the UC Davis Interdisciplinary Center for Plasma Mass Spectrometry (http://icpms.ucdavis.edu/). The numbers of atoms per cell were calculated for the observed quantities of each metal (parts per billion [nanograms per milliliter]) normalized to cell numbers estimated by colony enumerations.

### Autoaggregation flocculation assay.

*L. plantarum* B1.1 and B1.3 were incubated in 5 mL of cMRS, mMRS, or mMRS supplemented with 5 mM CaCl_2_·2H_2_O, 5 mM NaCl, 5 mM MnSO_4_·H_2_O, or 5 mM AlK_2_O_4_ (in triplicate) at 30°C for 24 h. The cultures were then vortexed vigorously for 10 s, and the OD_600_ was measured by collecting cells with a pipette tip placed 2 cm below the surface of the laboratory culture medium. The cultures were then incubated at 30°C, and the OD_600_ was measured again without disruption 24 h and 48 h later. Percent aggregation was calculated using the formula (1 − [final OD_600_/overnight OD_600_]) × 100, where final OD_600_ represents the optical density 2 cm below the surface at 48 h or 72 h and overnight OD_600_ represents the optical density 2 cm below the surface at 24 h from initial inoculation. At the end of the incubation period, the cultures were imaged to visualize the differences in the autoaggregation, flocculation phenotype.
