# Supplementary material for: Calcium Determines Lactiplantibacillus plantarum Intraspecies Competitive Fitness
Source: Appl Environ Microbiol. 2022 Jul 19;88(15):e00666-22. doi: 10.1128/aem.00666-22 (PMC9361822; doi:10.1128/aem.00666-22)
Supplement: Supplemental file 1 — Table S1 and Fig. S1 to S13. Download aem.00666-22-s0001.pdf, PDF file, 1.1 MB [file aem.00666-22-s0001.pdf]

Table S1. Genes present in *L. plantarum* WCFS1 and absent in *L. plantarum* B1.3

Location in WCFS1	Locus Tag	Gene Name	Product	Potential Function
99433 - 101717 (2,284)	lp_0113	*thiM*	Hydroxyethylthiazole kinase	Thiamine metabolism
	lp_0114	*thiD*	phosphomethylpyrimidine kinase & hydroxymethylpyrimidine kinase	
	lp_0115	*thiE*	thiamine-phosphate pyrophosphorylase	
981385 – 989646 (8,261)	lp_1081		hypothetical protein	Aromatic amino acid biosynthesis
	lp_1082		Malate/lactate dehydrogenase	
	lp_1083	*tkt2*	transketolase	
	lp_1084	*aroD1*	shikimate 5-dehydrogenase	
	lp_1085	*aroA*	phospho-2-dehydro-3-deoxyheptonate aldolase /chorismate mutase	
	lp_1086	*aroB*	3-dehydroquinate synthase	
1309330 – 1328902 (19,572)	lp_1427		nucleoside 2-deoxyribosyltransferase	
	lp_1430		glycosyltransferase	
	lp_1431		hypothetical membrane protein	
	lp_1433		phosphohydrolase	
	lp_1435		hypothetical membrane protein	
	lp_1436	*ribB*	riboflavin synthase, alpha chain	Riboflavin biosynthesis
	lp_1437	*ribA*	3,4-dihydroxy-2-butanone 4-phosphate synthase/ GTP cyclohydrolase II	
	lp_1438	*ribH*	riboflavin synthase, beta chain	
	lp_1439		nucleotidyltransferase superfamily protein	
	lp_1440		hypothetical protein	
	lp_1442		transcription regulator	
	lp_1443		transcription regulator	
	lp_1445	*npr1*	NADH peroxidase	
	lp_1446		Cell surface protein, CscB family	Unknown function cell surface proteins
	lp_1447		Cell surface protein precursor, CscD family	
	lp_1448		Cell surface protein precursor	
	lp_1449		Cell surface protein, CscB family	

	lp_1450		Cell surface protein, CscB family	
1502988 - 1521269 (25,963)	lp_1645		Alcohol dehydrogenase	
	lp_1648		ABC transporter, ATP-binding protein	
	lp_1649		ABC transporter, permease protein	
	lp_1652	*trpE*	Anthranilate synthase, component I	Tryptophan biosynthesis
	lp_1653	*trpG*	Anthranilate synthase, component II	
	lp_1654	*trpD*	Anthranilate phosphoribosyltransferase	
	lp_1655	*trpC*	Indole-3-glycerol-phosphate synthase	
	lp_1656	*trpF*	Phosphoribosylanthranilate isomerase	
	lp_1657	*trpA*	Tryptophan synthase, beta chain	
	lp_1658	*trpB*	Tryptophan synthase, alpha chain	
	lp_1659		Isochorismatase	
	lp_1660		Alcohol dehydrogenase, zinc-binding	
	lp_1662		Acetyltransferase	
	**lp_1663**		**Hypothetical membrane protein**[a]	
	**lp_1664**		**Zinc-dependent amidohydrolase**	
	lp_1665	*adh1*	Alcohol dehydrogenase, zinc-binding	
	lp_1667		Hypothetical membrane protein	
	lp_1668		Short-chain dehydrogenase/ oxidoreductase	
1535527 – 1548435 (12,908)	lp_1687	*rsgA*	Ribosome small subunit-dependent GTPase A	Ribosomal assembly
	lp_1688		Transcriptional regulator	
	lp_1689		Drug resistance transport protein	
	lp_1690		Hypothetical membrane protein	
	lp_1692		Hypothetical membrane protein	
	lp_1693		Transcriptional regulator	
	lp_1694		Hypothetical protein	
	lp_1695		Hypothetical membrane protein	

	lp_1696	*cfa1*	Cyclopropane-fatty-acyl-phospholipid synthase	Fatty acid biosynthesis
	lp_1697		Adherence protein	
	lp_1698		Antibiotic biosynthesis	
	lp_1699		Hypothetical protein	
	lp_1700		Hypothetical membrane protein	
	lp_1701		Nucleotide-binding protein, universal stress protein	
	lp_1702		Hypothetical membrane protein	
2595719 - 2606955 (11,236)	lp_2918	*ropB*	Transcriptional regulator	
	**lp_2919**	***pepR2***	**Prolyl aminopeptidase**	
	**lp_2920**		**Amino acid transport protein**	
	**lp_2921**		**Transport protein**	
	lp_2922	*npp*	Nucleotide pyrophosphatase	
	lp_2923		Lipase	
	lp_2924		Transcriptional regulator	
	lp_2925		Cell surface protein	
2639402 – 2658208 (18,806)	lp_2966		Multidrug ABC transporter	
	lp_2967		Transcriptional regulator	
	lp_2968		nitroreductase	
	lp_2969	*pts22CBA*	PTS system N-acetylglucosamine-specific EIICBA component	Carbohydrate transport – peptidoglycan recycling
	lp_2972		ABC transporter, ATP-binding protein	
	lp_2973		ABC transporter, permease protein	
	lp_2974		ABC transporter, substrate binding protein	
	lp_2975		Cell surface protein, CscC family	Unknown function cell surface proteins
	lp_2976		Cell surface protein precursor, CscD family	
	lp_2977		Cell surface protein precursor, CscA family	
	lp_2978		Cell surface protein, CscB family	
	lp_2979	*acuB-C*	Acetoin utilization protein, C-terminal fragment	Acetoin Utilization
	lp_2980	*acuB-N*	Acetoin utilization protein, N-terminal fragement	

	lp_2981	*livE*	Branched-chain amino acid ABC transporter, ATP-binding protein	Branched-chain amino acid transport
	lp_2982	*livD*	Branched-chain amino acid ABC transporter, ATP-binding protein	
	lp_2983	*livC*	Branched-chain amino acid ABC transporter, permease protein	
	lp_2984	*livB*	Branched-chain amino acid ABC transporter, permease protein	
	lp_2985	*livA*	branched-chain amino acid ABC transporter, substrate binding protein	
3024823 – 3031219 (6,396)	lp_3001		Cell surface protein precursor	
	lp_3002		Hypothetical membrane protein	
	lp_3003		Metallo-phosphoesterase	
	lp_3004		Hypothetical protein	
	lp_3006		Transcriptional regulator	
	lp_3008	*pts23A*	PTS system, cellobiose-specific EIIA component	Carbohydrate metabolism
	lp_3009	*pts23B*	PTS system, cellobiose-specific EIIB component	
	lp_3010	*pts23C*	PTS system, cellobiose-specific EIIC component	
	lp_3011	*pbg6*	6-phospho-beta-glucosidase	
	lp_3012		NAD-dependent epimerase	
	lp_3013		Transcriptional regulator	
	lp_3014		Extracellular transglucosylase	
	lp_3015		Extracellular transglucosylase	
	lp_3016		Hypothetical membrane protein	
	lp_3017		Hypothetical protein	
	lp_3018		ABC transporter, substrate binding protein	
	lp_3019		Extracellular protein, membrane-anchored	
2860144 - 2874309 (14,165)	lp_3209		Cystine ABC transporter, substrate binding protein	Cystine amino acid transport
	lp_3210		Cystine ABC transporter, permease protein	
	lp_3211		Cystine ABC transporter, ATP-binding protein	

	lp_3214		Cystathionine ABC transporter, substrate binding protein	
	lp_3215		Hypothetical protein	
	lp_3216		Transcriptional regulator	
	lp_3217		Hypothetical membrane protein	
	lp_3218		extracellular protein, membrane-anchored	
	lp_3219	*pts26BCA*	PTS system, sucrose-specific EIIBCA component	Carbohydrate metabolism
	lp_3220		sucrose/trehalose-6-phosphate hydrolase	
	lp_3221		Transcriptional regulator, LacI family, sucrose related	
	lp_3223		Hypothetical membrane protein	
	lp_3224		ABC transporter, permease protein	
	lp_3225		ABC transporter, ATP-binding protein	
3024823 – 3031219 (6,393)	lp_3412		Cell surface protein, CscB family	Unknown function cell surface proteins
	lp_3413		Cell surface protein precursor, CscA family	
	lp_3414		Cell surface protein, CscB family	
	lp_3415		Transcriptional regulator	
3072533 – 3085235 (12,702)	lp_3468	*lacS*	PTS regulated carbohydrate transporter	Carbohydrate metabolism
	lp_3469	*lacA*	Beta-galactosidase I	
	lp_3470	*lacR*	Transcriptional regulator	
	lp_3471	*ram1*	Alpha-L-rhamnosidase	Carbohydrate metabolism
	lp_3472	*ramP1*	Disaccharide transporter	
	lp_3473	*ram2*	Alpha-L-rhamnosidase	
	lp_3474	*ramP2*	Disaccharide transporter	
	lp_3476	*ramR*	Transcriptional regulator	

[a] Bold incdicates genes that are absent in both *L. plantarum* B1.1 and B1.3 compared to WCFS1

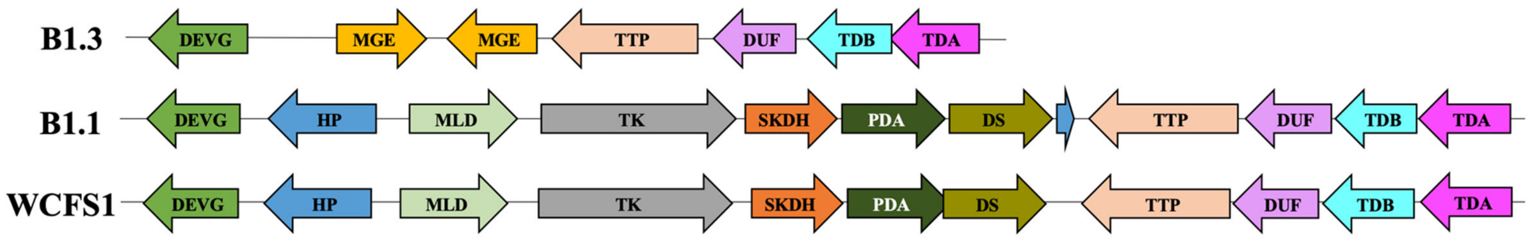

Fig. S1. *L. plantarum* B1.3 lacks shikimate pathway genes required for aromatic amino acid biosynthesis. Genome organization of shikimate pathway genes in *L. plantarum* strains. DEVG: fatty acid-binding protein, DegV family (lp_1079), HP: hypothetical protein (lp_1081), MLD: malate/lactate dehydrogenase (lp_1082), TK: transketolase (*tkt2*; lp_1083), SKDH: shikimate 5-dehydrgonase (aroD1; lp_1084), PDA: phosphor-2-deoxyheptonate aldolase/ chroismate mutase (aroA; lp_1085), DS: 3-dehydroquinate synthase (aroB; lp_1086). TTP: tartate transport protein (ttpD; lp_1087), DUF: hypothetical protein, DUF59 family (lp_1088), TDB: L(+)-tartrate dehydrates, subunit B (ttdB; lp_1089), TDA: L(+)-tartrate dehydrates, subunit A (ttdA; lp_1090), MGE: mobile genetic element.

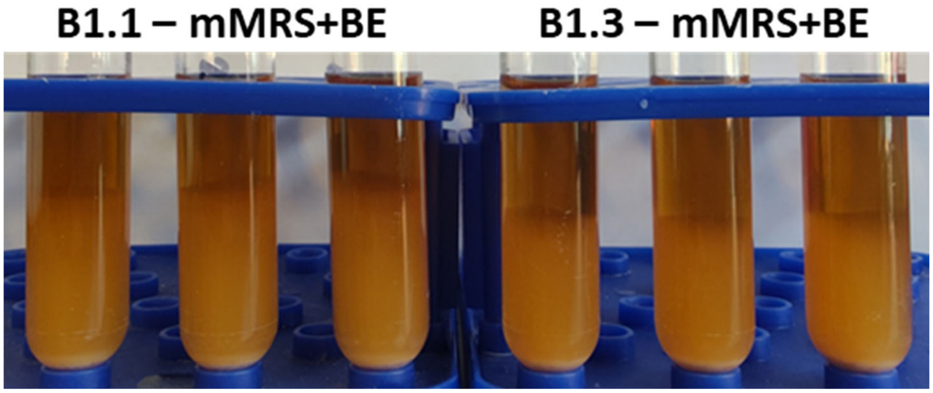

Fig. S2. Auto-aggregation of *L. plantarum* B1.1 and B1.3 in mMRS supplemented with beef extract (BE). *L. plantarum* B1.3 and B1.1 cultures were imaged after incubation at 30 °C for 72 h.

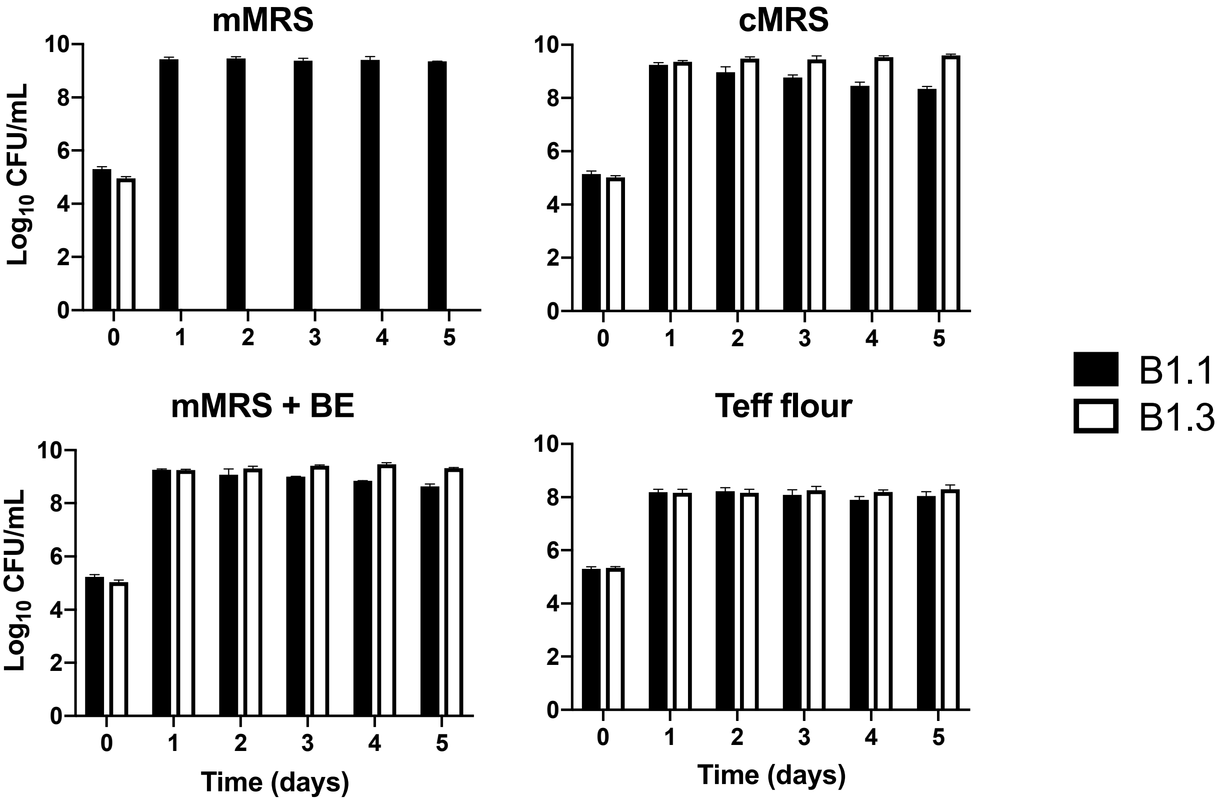

Fig. S3. Numbers of *L. plantarum* B1.1 and B1.3 in mMRS, cMRS, mMRS supplemented with beef extract, and a teff flour suspension. Equal numbers of *L. plantarum* B1.1 and B1.3 (10^5 CFU/ml) were co-inoculated in mMRS, cMRS, mMRS supplemented with beef extract (8 g/L), or teff flour mixed with PBS and incubated at 30 ˚C for 24 h. A total of 50 µl was transferred into fresh medium (constituting 1% of the final volume) on each of the subsequent five days. The avg ± stdev of three replicate cultures are shown.

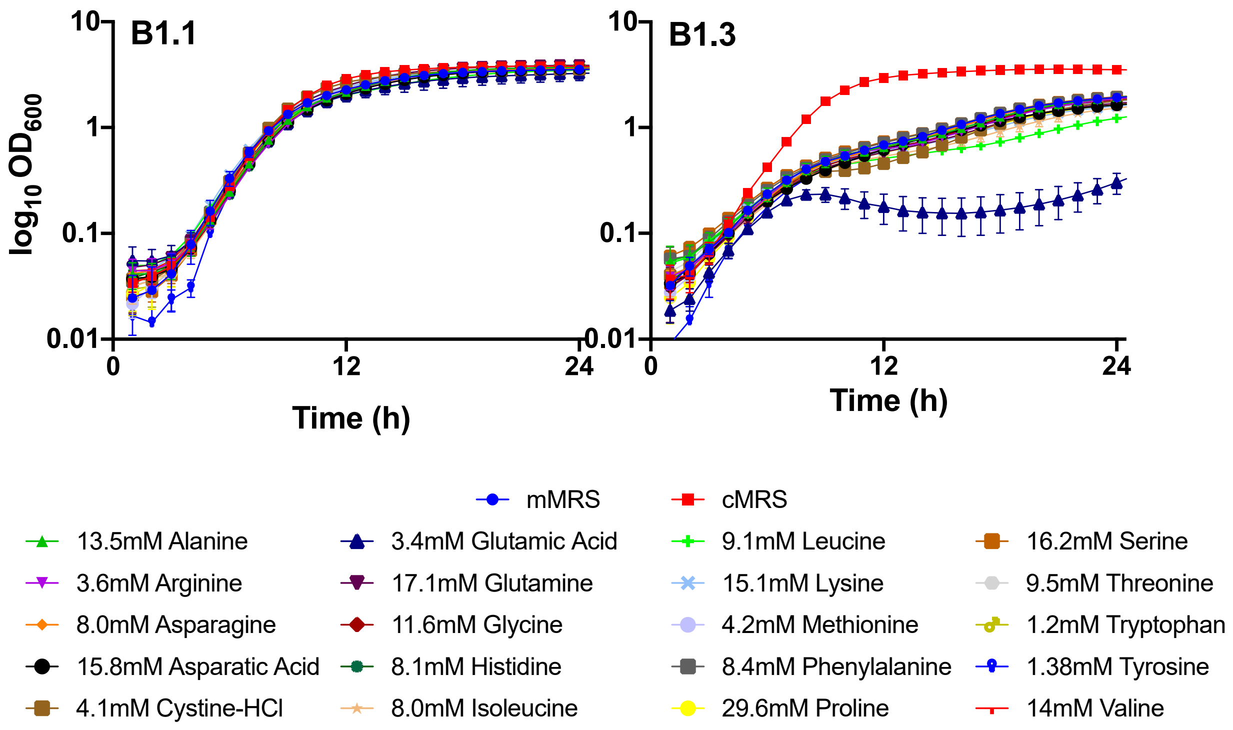

Fig. S4. Growth of *L. plantarum* B1.1 and B1.3 in mMRS supplemented with different amino acids. *L. plantarum* B1.1 and B1.3 were inoculated into mMRS supplemented with single amino acids and incubated at 30 °C for 24 h. Results shown are representative avg ± stdev OD_{600} of duplicate experiments with three independent cultures.

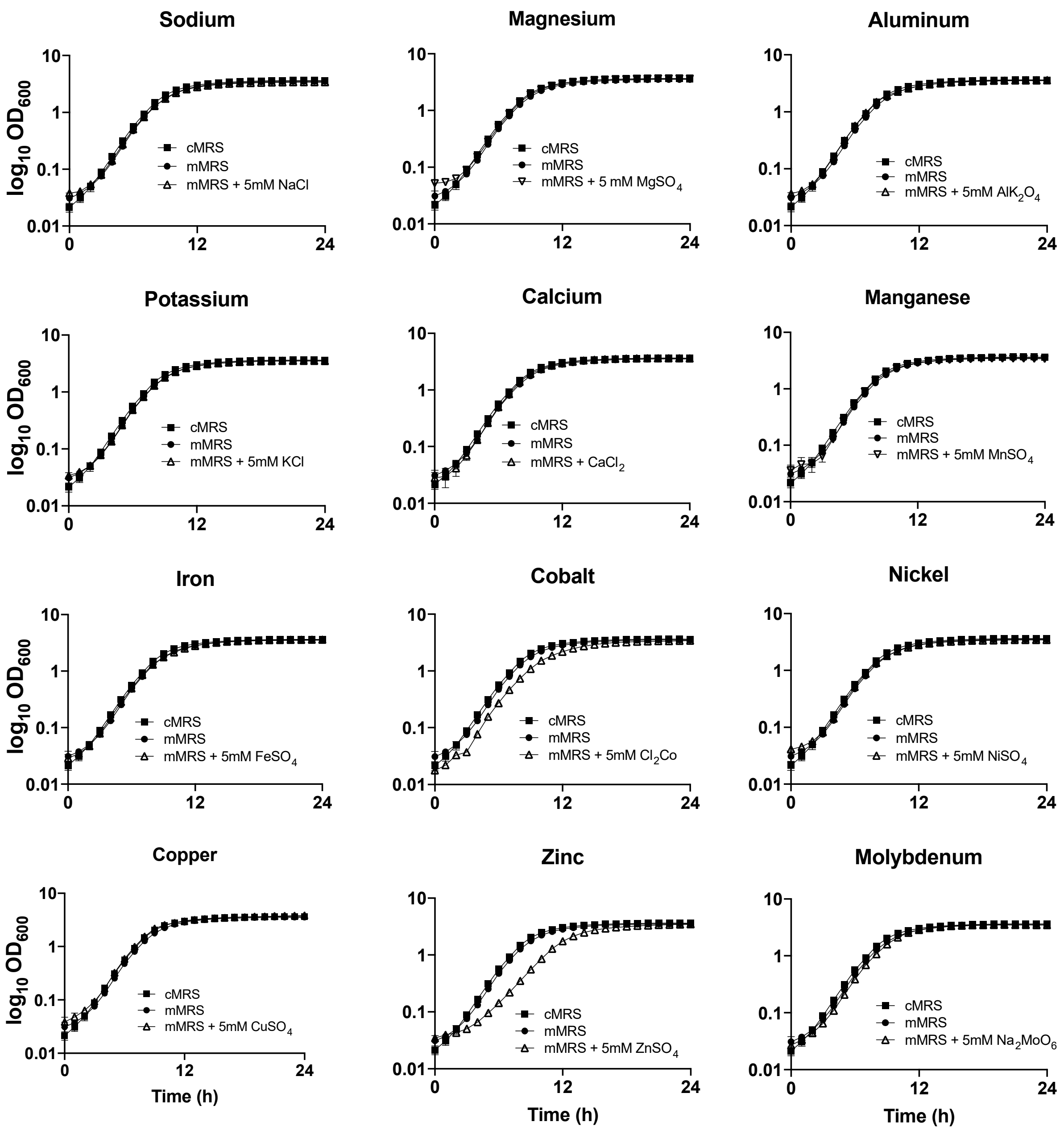

Fig. S5. Growth of *L. plantarum* B1.1 in mMRS supplemented trace metals. *L. plantarum* B1.1 were inoculated into mMRS containing 5mM of individual trace metals and incubated at 30 °C for 24 h. Results shown are representative avg ± stdev OD_{600} of duplicate experiments with three independent cultures.

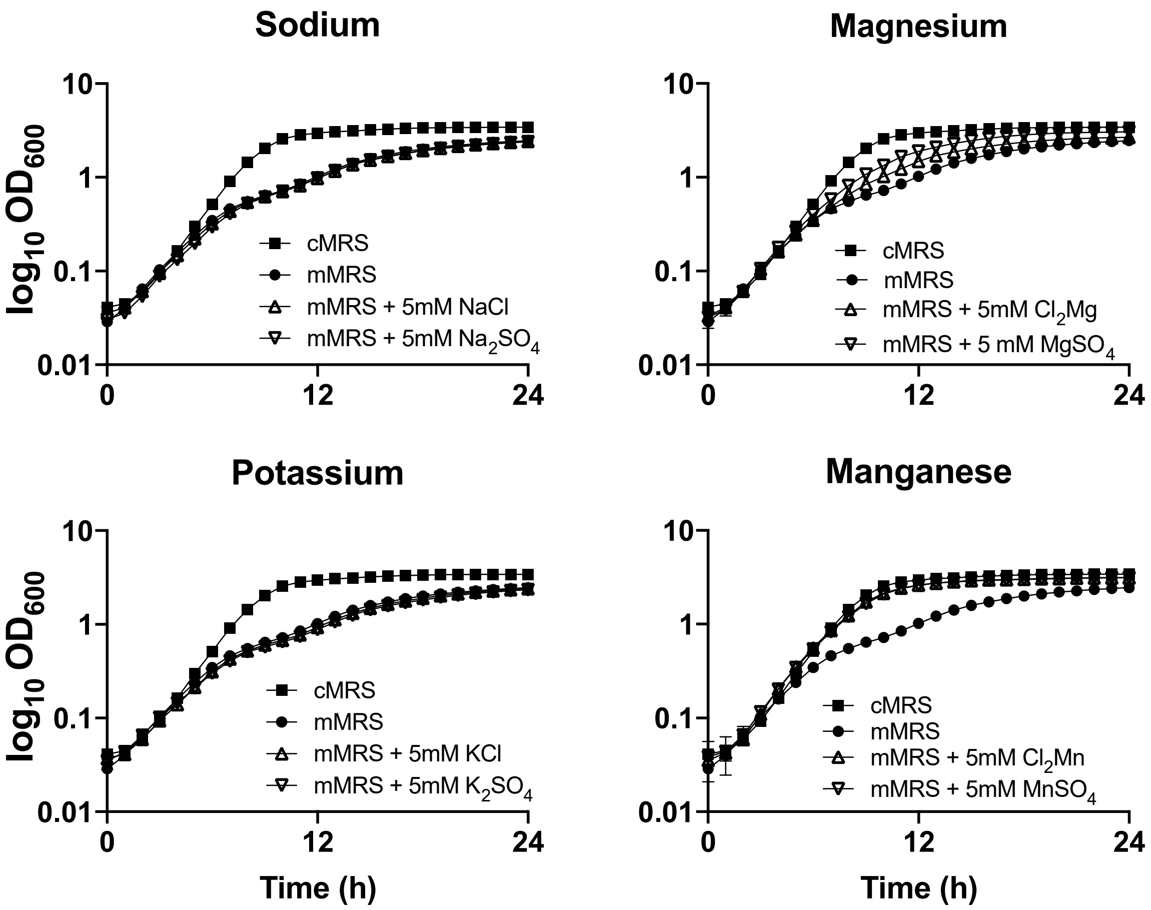

Fig. S6. Comparison of growth of *L. plantarum* B1.3 in mMRS supplemented in the sulfide or chloride form of the metal ion. *L. plantarum* B1.3 were inoculated into mMRS containing 5mM of individual trace metals and incubated at 30 °C for 24 h. Results shown are representative avg ± stdev OD_{600} of duplicate experiments with three independent cultures.

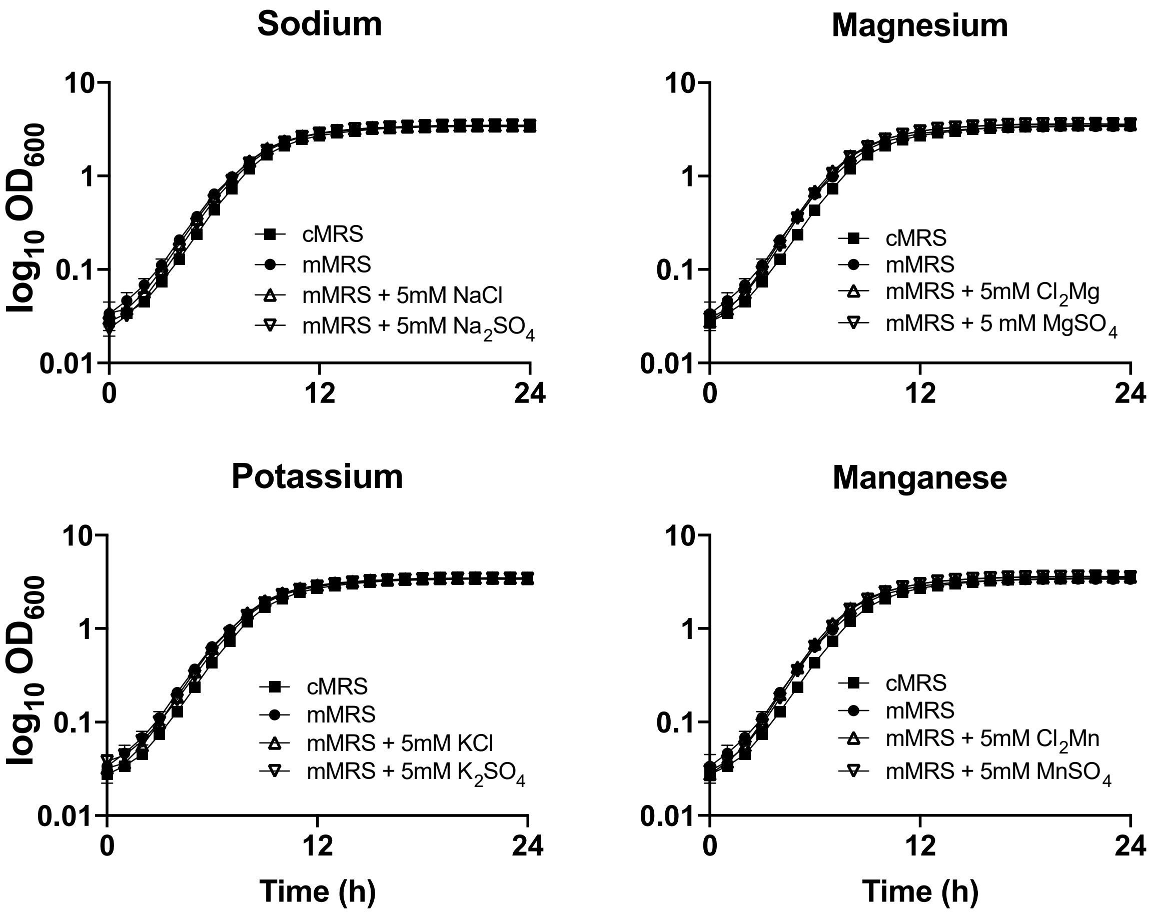

Fig. S7. Comparison of growth of *L. plantarum* B1.1 in mMRS supplemented in the sulfide or chloride form of the metal ion. *L. plantarum* B1.1 were inoculated into mMRS containing 5mM of individual trace metals and incubated at 30 °C for 24 h. Results shown are representative avg ± stdev OD_{600} of duplicate experiments with three independent cultures.

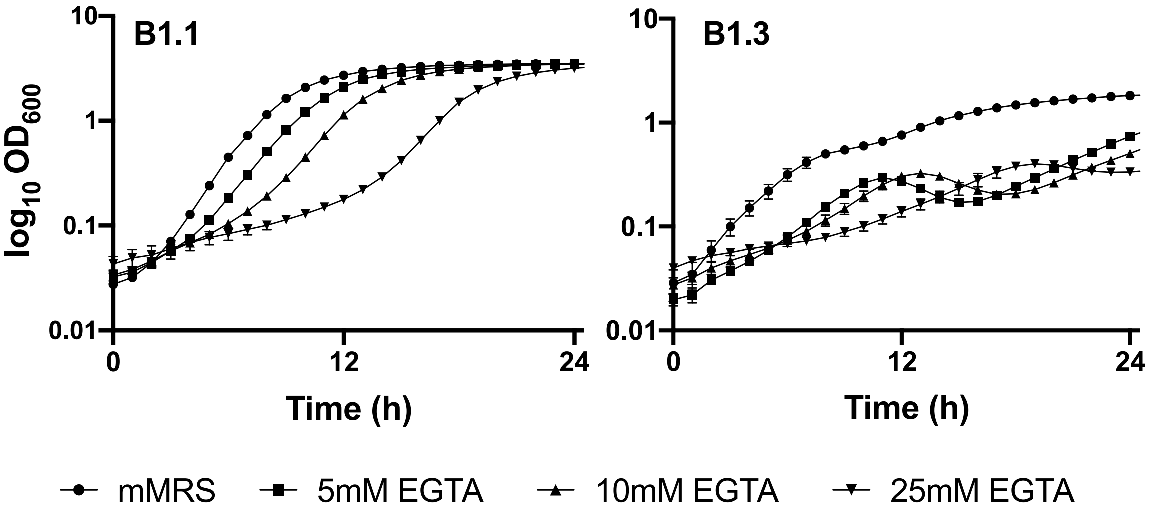

Fig. S8. Growth of *L. plantarum* in mMRS supplemented with different concentrations of EGTA. *L. plantarum* B1.1 and B1.3 were incubated at 30 °C for 24 h. Results shown are representative avg ± stdev OD_{600} of duplicate experiments with three independent cultures.

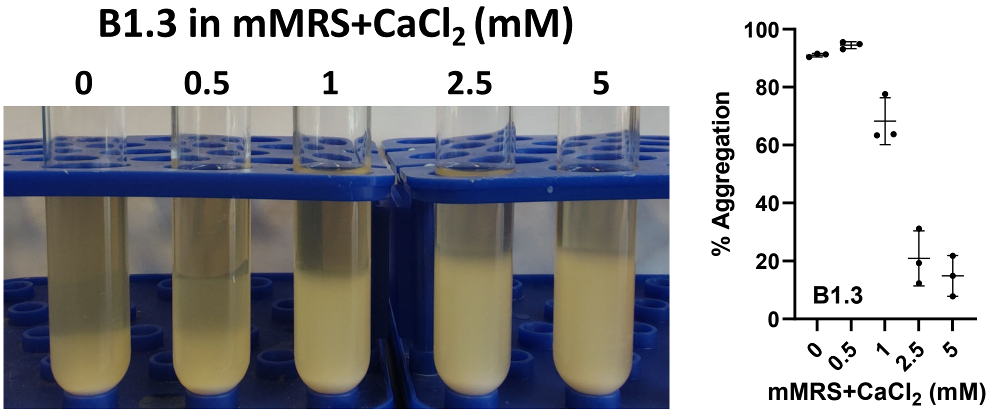

Fig. S9. Auto-aggregation of *L. plantarum* B1.3 in mMRS supplemented with different concentrations of $CaCl_2$. *L. plantarum* B1.3 cultures grown in triplicate were imaged after incubation at 30 °C for 72 h. The % aggregation is shown as mean ± stdev between triplicates. Representative cultures are shown.

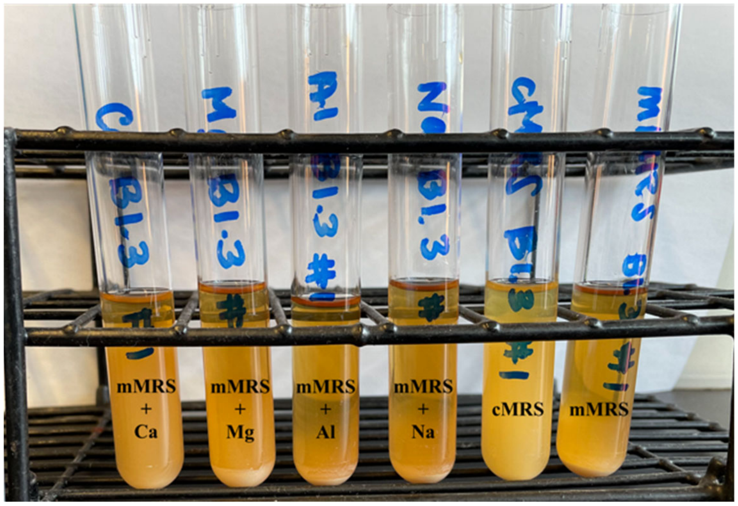

Fig. S10. Auto-aggregation of *L. plantarum* B1.3 in mMRS, cMRS, and mMRS supplemented with 5mM of $CaCl_2$, $MgSO_4$, AlK_2O_4, or NaCl. *L. plantarum* B1.3 cultures grown in triplicate were imaged after incubation at 30 °C for 72 h. Representative cultures are shown.

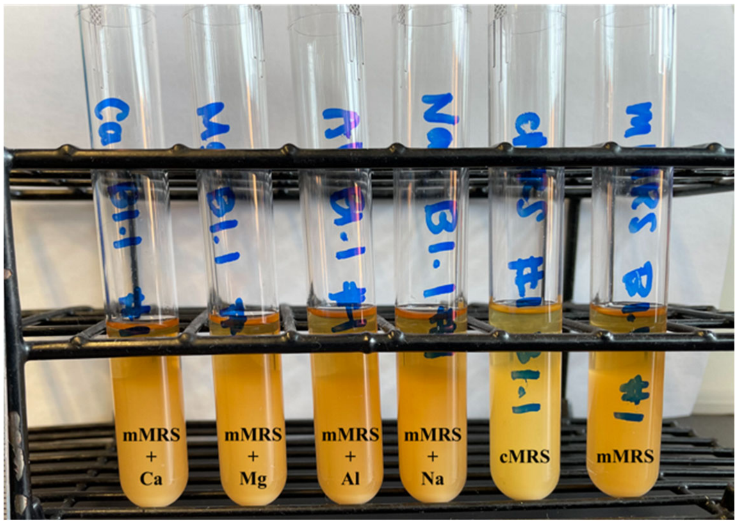

Fig. S11. Auto-aggregation of *L. plantarum* B1.1 in mMRS, cMRS, and mMRS supplemented with 5mM of $CaCl_2$, $MgSO_4$, AlK_2O_4, or NaCl. *L. plantarum* B1.1 cultures grown in triplicate were imaged after incubation at 30 °C for 72 h. Representative cultures are shown.

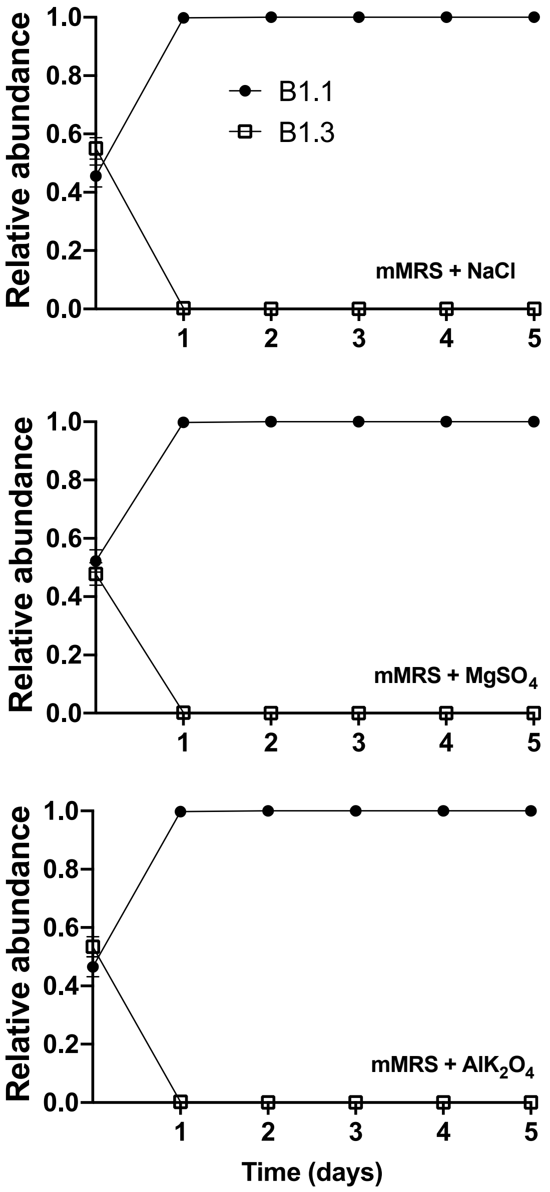

Fig. S12. Competitive fitness of *L. plantarum* B1.1 and B1.3 in mMRS 5mM of NaCl, $MgSO_4$, or AlK_2O_4. Equal numbers of *L. plantarum* B1.1 and B1.3 (10^5 CFU/ml) were co-inoculated in mMRS 5mM of NaCl, $MgSO_4$, or AlK_2O_4. A total of 50 µl was transferred into fresh medium (constituting 1% of the final volume) on each of the subsequent five days. The avg ± stdev of three replicate cultures is shown.

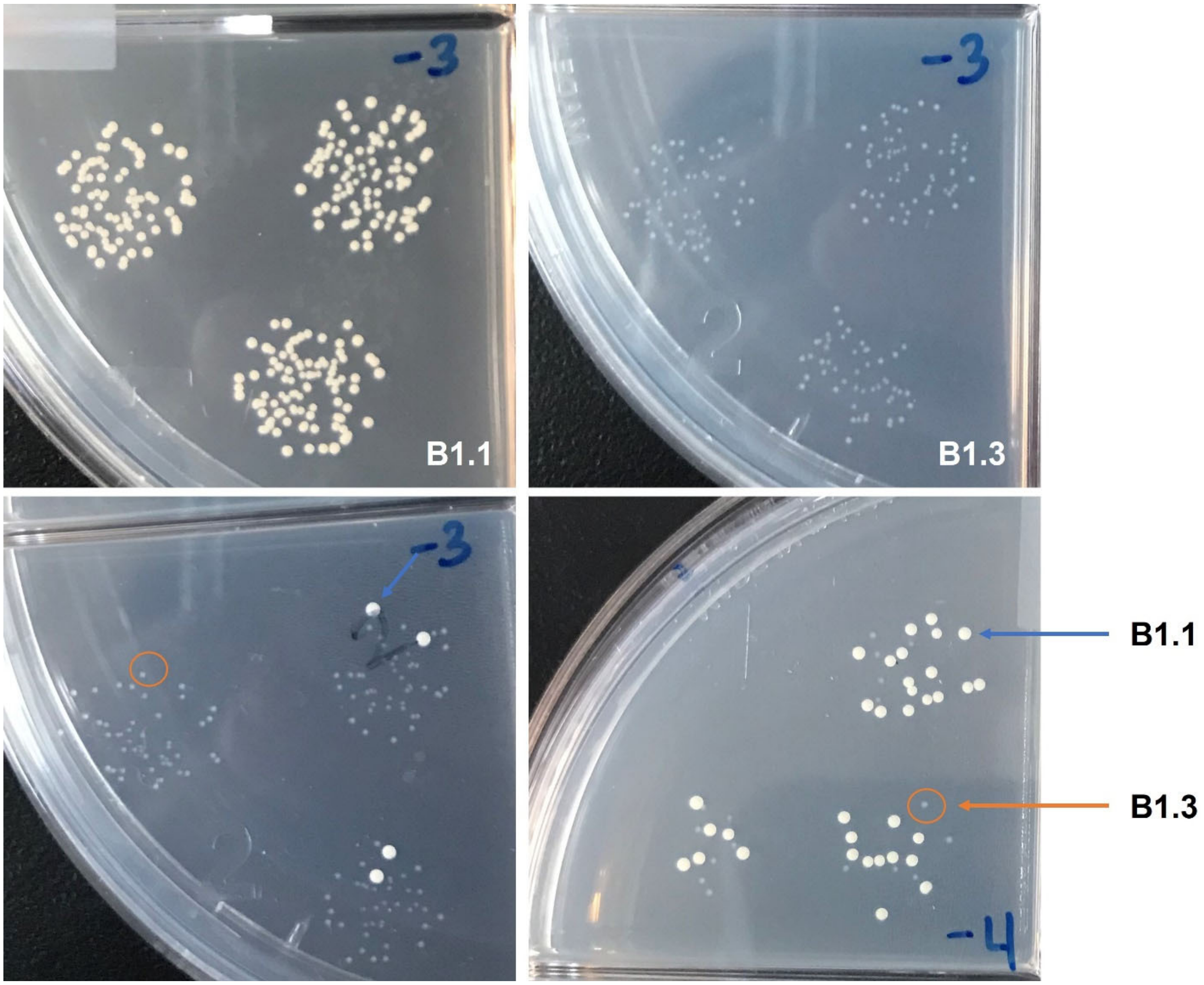

Fig. S13 *L. plantarum* B1.1 and B1.3 colony morphology on mMRS-sucrose. *L. plantarum* B1.1 and B1.3 grown separately (top two panels) and then mixed in different proportions (bottom panels) numbers before plating on mMRS-sucrose. The plates were incubated at 30°C for 24 h. Representative agar plates are shown.
